# Identification of isomeric cyclo(leu-pro) produced by *Pseudomonas sesami* BC42 and its differential antifungal activities against *Colletotrichum orbiculare*

**DOI:** 10.3389/fmicb.2023.1230345

**Published:** 2023-08-10

**Authors:** Jiwon Kim, Jin-Cheol Kim, Mee Kyung Sang

**Affiliations:** ^1^Division of Agricultural Microbiology, National Institute of Agricultural Sciences, Rural Development Administration, Wanju, Republic of Korea; ^2^Department of Agricultural Biology, College of Agricultural and Life Sciences, Jeonbuk National University, Jeonju-si, Republic of Korea; ^3^Department of Agricultural Chemistry, Institute of Environmentally Friendly Agriculture, College of Agriculture and Life Sciences, Chonnam National University, Gwangju, Republic of Korea

**Keywords:** antifungal, anthracnose, biocontrol, cyclo(l-leu-l-pro), *Pseudomonas*

## Abstract

*Pseudomonas* spp. produce various antimicrobial substances, including cyclic peptides, which have been shown to suppress fungal pathogens. In a previous study, *Pseudomonas sesami* BC42 was selected to control anthracnose caused by *Colletotrichum orbiculare* in cucumber plants, and the bioactive extract of strain BC42 inhibited fungal growth and development. In this work, preparative thin-layer chromatography was conducted to identify the antifungal compounds in the extract of strain BC42, and the portion of the extract that exhibited antifungal activity was further analyzed by gas chromatography–mass spectrometry. Three different isomers of the cyclic dipeptide, cyclo(Leu-Pro), were identified: cyclo(l-Leu-l-Pro), cyclo(d-Leu-d-Pro), and cyclo(d-Leu-l-Pro). Among these, 100 μg/mL of cyclo(l-Leu-l-Pro) significantly and more effectively inhibited the germination of conidia and appressorium formation and reduced leaf lesion size caused by *C. orbiculare*, relative to the control; cyclo(d-Leu-d-Pro) significantly reduced conidia germination and lesion occurrence, however, cyclo(d-Leu-l-Pro) did not exhibit antifungal activity. Therefore, the cyclo(l-Leu-l-Pro) and cyclo(d-Leu-d-Pro) derived from *P. sesami BC42* may be a promising candidate for biocontrol applications in agriculture.

## 1. Introduction

Plant diseases affect the agricultural industry and result in significant economic losses and reduced product quality. Disease control relies primarily on synthetic chemical pesticides. To reduce the use of chemical pesticides, various biological control options using antagonistic microorganisms or microbial pesticides have been developed as effective alternatives, owing to their reduced toxicity and higher biodegradability in natural environments. Biological control continues to grow in popularity and is becoming a preferred alternative, with the aim of reducing the use of chemicals in sustainable agriculture. Plant growth-promoting rhizobacteria are important bacterial communities in the rhizosphere that respond to soil-borne pathogens and serve as biocontrol agents (Backer et al., 2018).

*Pseudomonas* is one of the most abundant genera of soil-inhabiting bacteria on the surfaces of seeds and roots, enhancing plant growth and suppressing plant diseases (Mercado-Blanco and Bakker, 2007; Dimkić et al., 2022). *Pseudomonas* exhibits several traits associated with biocontrol and produces various antimicrobial substances, including hydrogen cyanide, phenazine-1-carboxylic acid, 2,4-diacetylphloroglucinol, pyrrolnitrin, pyoluteorin, and cyclic lipopeptides, which have been shown to suppress fungal pathogens. Additionally, the siderophores produced by *Pseudomonas* can make iron unavailable for the growth of fungal pathogens *via* chelation, while the biosurfactants and hydrolytic enzymes of *Pseudomonas* can support biocontrol. Together, these traits make *Pseudomonas* an important and promising candidate for applications in agriculture and medicine (Shtark et al., 2003; Weller, 2007; Patil et al., 2017; Keswani et al., 2019; Raio and Puopolo, 2021).

Cyclic dipeptides (CDPs), formed by the inner cyclization of two amino acid amides, are usually synthesized by prokaryotic and eukaryotic cells using cyclodipeptide synthases or non-ribosomal peptide synthases (Rhee, 2002). Several cyclic peptides with different structures have been characterized to act against pathogenic fungi and bacteria (Lee and Kim, 2015). Park et al. (2012) reported that cyclo(l-Pro-l-Val) from *P. aurantiaca* IB5-10 exhibits antifungal activity against *Rhizoctonia solani*. Cyclo(l-Pro-d-Ile) and cyclo(l-Pro-l-Phe) from *Escherichia coli* interfere with the expression levels of certain pathogenicity contributors of *Ralstonia solanacearum* (Song et al., 2018). The mode of action of cyclic dipeptides in controlling plant diseases is not fully understood; however, several mechanisms are involved, including disruption of fungal cell membranes, inhibition of the activity of enzymes involved in fungal cell wall synthesis, and induction of plant defense responses against fungal pathogens (Houston et al., 2002; Song et al., 2017; Balachandra et al., 2021).

Anthracnose, caused by the fungus *Colletotrichum orbiculare*, is a common disease that affects a wide range of plants, including cucumbers, and may significantly damage cucumber crops (Agrios, 2005). The symptoms of anthracnose in cucumbers typically include small water-soaked lesions on the leaves, stems, and fruit of the plant. As the disease progresses, the lesions may enlarge and become sunken, with a brown or black color. The fruits may also develop dark spots and become distorted or misshapen. In severe cases, the leaves may turn yellow and drop from the plant, and the fruit may rot and become unusable. This disease can spread through water, wind, and contaminated tools or equipment, making effective disease-control measures a challenge (Damm et al., 2013; Kubo and Takano, 2013).

Infection with *C. orbiculare* begins with the germination of fungal conidia on the plant surface. Once on the surface, the conidia germinate and produce germ tubes, which then penetrate the plant tissue using enzymes to degrade the cell wall and produce the appressorium. After entering the plant, the fungus grows and reproduces, damaging the plant tissue and leading to the development of anthracnose disease symptoms (Osherov and May, 2001; Kubo and Takano, 2013). Therefore, to prevent *C. orbiculare* infection, it is important to disrupt the fungal life cycle by limiting fungal growth and development, including spore production, mycelial growth, and appressorium formation. In a previous study, we selected *Pseudomonas* sp. BC42 as a biocontrol agent against *C. orbiculare*, as it exhibited antifungal activity against certain bioactive compounds extracted using ethyl acetate (Kim et al., 2022). The strain BC42 was identified as *Pseudomonas sesami* based on 16S rRNA sequencing analysis (Kim et al., 2022).

In the present work, we aimed to: (1) identify the antifungal compounds derived from strain BC42 and (2) evaluate contribution of the identified compounds to the antifungal activity against *C. orbiculare,* as well as disease suppression in cucumber plants. Our investigations returned three forms of cyclo(Leu-Pro) from the BC42-extract, which demonstrated differential biocontrol activities in *in vitro* and detached leaf assays.

## 2. Materials and methods

### 2.1. Isolation of bioactive metabolites by thin-layer chromatography

The BC42 strain was cultured on tryptic soy broth medium (TSB, Difco, USA) for 72 h in a shaker incubator at 160 rpm at 28°C. The filtrate of BC42 culture broth was obtained after centrifugation at 6,000 rpm for 20 min and filtered through a PES filter (0.45 μm, GVS, Italy). The filtered culture broth was mixed with an equal volume of ethyl acetate (HPLC-grade, 1:  1 (v/v), Merck, Germany), followed by extraction using a separating funnel (Kim et al., 2022). The separated fractions were dried over Na_2_SO_4_ and evaporated using a rotary vacuum evaporator (Eyela Co. Ltd., Japan). The dried extract was dissolved in methanol (HPLC grade; Merck, Germany), and a stock concentration (10 mg/mL) was prepared. Thin-layer chromatography (TLC) was performed to analyze the development patterns of antifungal metabolites. Five microliters of ethyl acetate extracts were spotted onto TLC plates (HPTLC Silica gel 60 F_254_, Merck, Germany). The TLC plate was developed in a glass chamber containing chloroform and methanol (97:3 v/v) as the mobile phases. The plate was dried and visualized under short (254 nm) and long (366 nm) light wavelengths. The bioautography method was used to determine antifungal activity against *C. orbiculare* (Wedge and Nagle, 2000; Dewanjee et al., 2015). The solvent used to develop the TLC plate was removed, and the plate was sterilized with UV light on a clean bench. Potato dextrose agar (PDA, Difco, USA) (0.8% agar) was amended with a spore suspension of *C. orbiculare* (final concentration, 5 × 10^6^ conidia/mL per medium), and a developed TLC plate was placed on the medium. The clear zone of inhibition was observed after 7 days of incubation at 28°C and compared with the band position of the TLC plate. The crude extracts obtained by preparative TLC (prep-TLC) were dissolved in methanol (100 μg/mL) and used for determining their minimum inhibitory concentration (MIC) against *C. orbiculare*, according to the microdilution assay. Prep-TLC was performed to purify and identify the antifungal compounds for further gas chromatography–mass spectrometry (GC–MS) analysis. Prep-TLC was carried out on PLC Silica gel 60 F_254_ (0.5 mm, Merck, Germany) using a mobile phase of chloroform: methanol (97:3). The metabolites extracted by prep-TLC were eluted using HPLC-grade acetone (Merck, Germany). The extract was evaporated using a rotary vacuum evaporator and stored at −20°C until use.

### 2.2. Identification of bioactive metabolites by GC–MS analysis

The GC–MS analysis of prep-TLC extracts was performed using a GC–MS QP2010 system (Shimadzu, Japan) equipped with a DB-5MS (30 m × 0.25 mm, film thickness 0.25 μm) capillary column (Agilent Technologies, USA). Helium was used as the carrier gas at a flow rate of 1.2 mL/min. The oven temperature was 170°C for 2 min, raised to 280°C at a rate of 30°C/min, and then held for 33 s. Finally, the temperature increased by 2°C/min to 300°C and was maintained for 14 min. Samples (1,000 μg/mL) of 1 μL were injected in split mode (ratio 5:1). Ion source and interface temperatures were set at 200°C and 250°C, respectively. Identification of metabolites was performed by the NIST/EPA/NIH Mass Spec Library (version 2.0), comparing the mass spectra of various substances.

### 2.3. Assessment of conidia germination and appressorium formation of *Colletotrichum orbiculare*

The conidia of *C. orbiculare* were harvested after 7 days of incubation on PDA and filtered through six layers of sterile cheesecloth to remove hyphal residues. For the germination and appressorium formation assay, three forms of cyclo(Leu-Pro), including cyclo(l-Leu-l-Pro), cyclo(d-Leu-d-Pro), and cyclo(d-Leu-l-Pro), were purchased from the Peptron (Daejeon, South Korea); an ethyl acetate extract of BC42 (final concentration of 100 μg/mL) and the cyclo(Leu-Pro) (1, 10, and 100 μg/mL, diluted using sterile distilled water (SDW)) were mixed (1:1) with a conidial suspension (2 × 10^5^ spores/mL), and 50 μL of the mixture was placed on a glass slide and incubated at 25°C for 24 h while maintaining 100% relative humidity. SDW was used as the negative control. A total of 50–100 conidia were examined for germination assessment, and up to 50 germinated conidia were examined for each assessment of appressorium formation. The following formula was used: number of germinated conidia/total conidia and number of appressorium/germinated conidia × 100 (Ghajar et al., 2006; Liu et al., 2008). Tests were performed using data from three independent experiments with six replicates (n = 18).

### 2.4. Detached leaf assay of *Colletotrichum orbiculare*

Cucumber seeds (Jo-eun Baekdadagi, Heongnong, South Korea) were sown in a 10 cm plastic pot filled with a commercial potting mixture (Baroker, South Korea) and grown in a greenhouse (25°C ± 5°C, day temperature; 20°C ± 5°C, night temperature). The first leaves of cucumber seedlings at the 1–2 leaf stage were used for the detached leaf assays. The conidia of *C. orbiculare* were harvested seven days after incubation on PDA. Ethyl acetate extract and cyclic peptide were prepared at concentrations of 100 μg/mL and 1–100 μg/mL, respectively. Distilled water was used as the control. The tested metabolites were mixed (1:1) with a conidial suspension (1 × 10^6^ spores/mL) and dropped onto the abaxial surface of the first leaf. Detached leaves were placed on a moistened square dish (20 × 20 cm, SPL Life Science, South Korea) and incubated in a growth chamber at 28°C (16 h light/day). The area of lesions caused by *C. orbiculare* on the first leaf at 7 days post-inoculation (dpi) was calculated using the following formula: S = π × a × b (where a and b are the major and minor radii, respectively). The experiments were independently conducted three times with five plants each (n = 15).

### 2.5. Statistical analysis

Statistical analyses were performed using R studio (version 1.4.1106) software (R studio, United States) (Team, R, 2015) on R platform (Team, RDC, 2009). Bartlett’s test was performed to assess the equality of variances for a variable calculated between groups. Differences among means were analyzed using analysis of variance (ANOVA) in the “agricolae” package (de Mendiburu, 2019). For germination, appressorium, and lesion area data, two-way (two factors: treatment and concentration) ANOVA was performed. Pearson’s correlation (*r*) was used to demonstrate the relationship between lesion size and the germination or appressorium formation treated by three types of CDPs using the “psych” package. Significant differences between treatments were evaluated using Tukey’s HSD test at *p* < 0.05.

## 3. Results

### 3.1. Identification of active compounds from the BC42-extract using GC–MS

**Figure 1:**
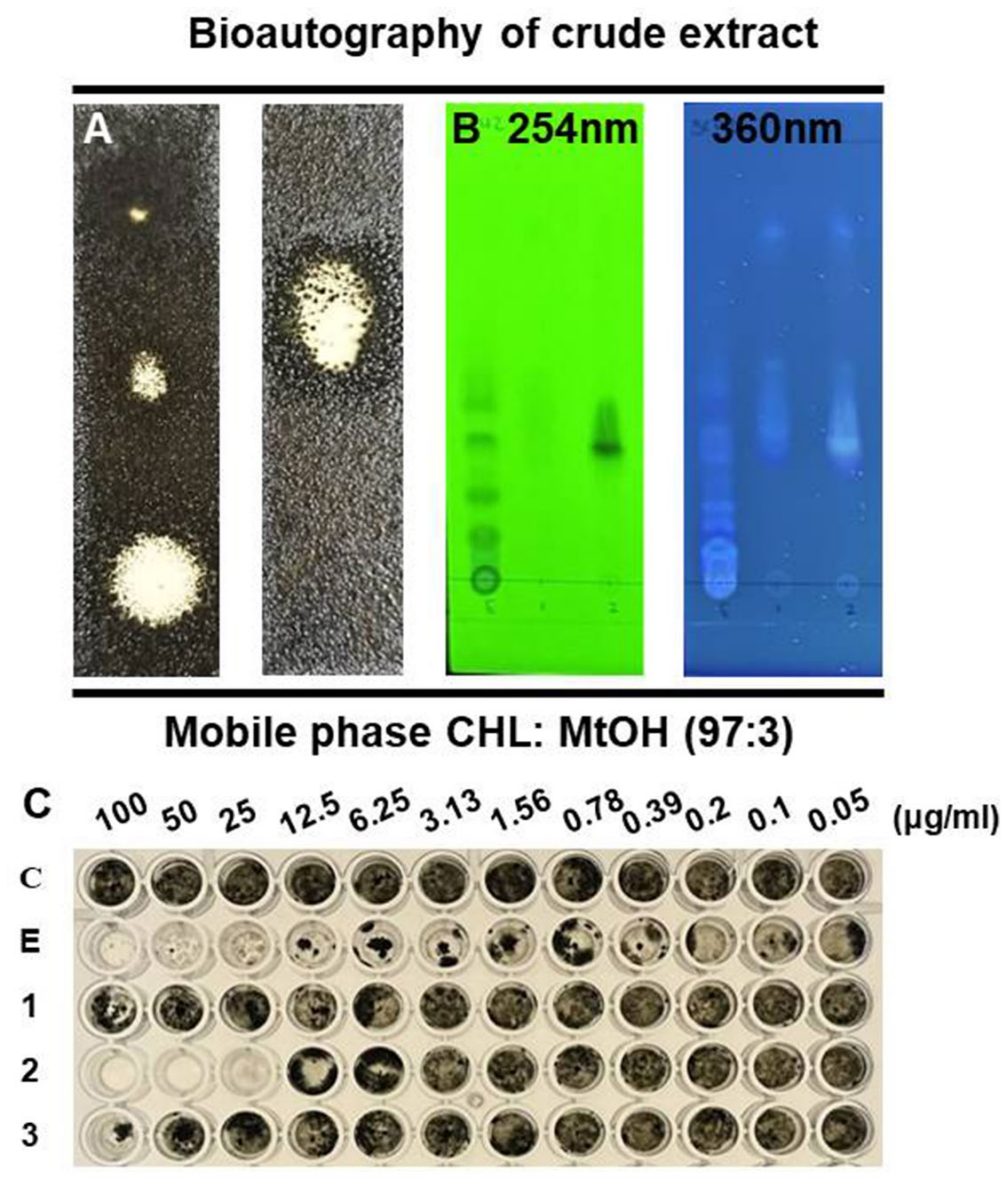
TLC profile of the crude extract from *P. sesami* BC42. **(A)** Bioautography, **(B)** viewed under UV light, and **(C)** minimum inhibitory concentration of crude extract. Symbols used: C; control, E; ethyl acetate extract of BC42, 1; line 1, 2; line 2, 3; line 3.

**Figure 2:**
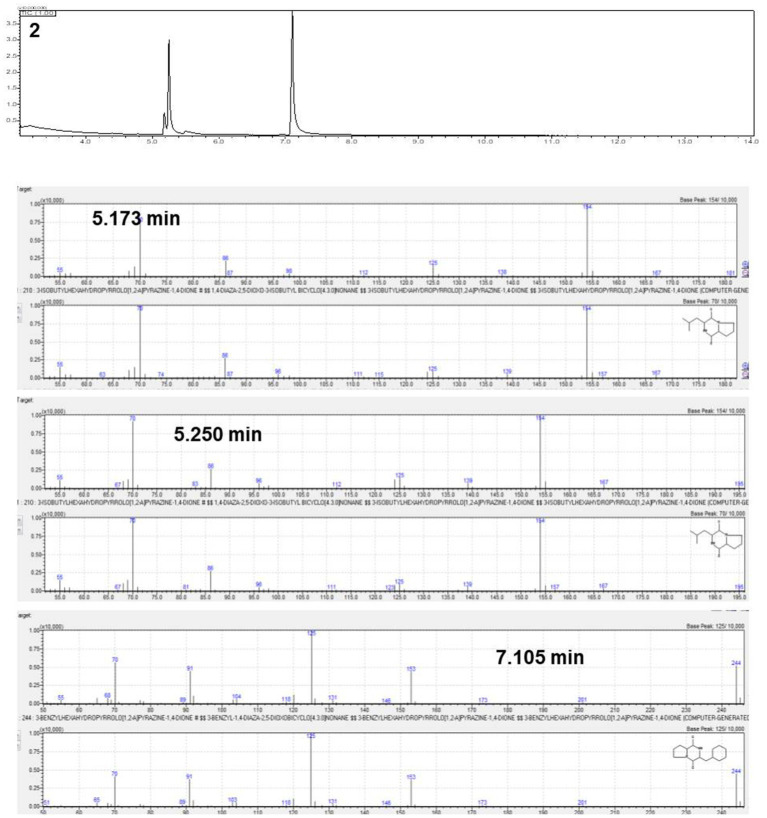
Gas chromatography–mass spectrometry (GC–MS) analysis of the number 2 fraction.

The active antifungal substances in the ethyl acetate extract of BC42 were explored by bioautography after TLC (Figure 1A). It was speculated that the active compounds were located at Rf 0.53 when developed using a mobile solvent of C:M = 97:3 (Figure 1B). Lines 1–3 around Rf 0.53 were scraped off and dissolved in acetone. The growth inhibition effect was observed at line 1 and line 2, with concentration ranges of 100–25 μg/mL and 100–6.25 μg/mL, respectively (Figure 1C). A GC–MS analysis was performed to predict the possible antifungal compounds in *P. sesami* BC42, which identified cyclo(Leu-Pro), with a retention time of 5.18 min (Figure 2).

### 3.2. Differential antifungal activity of isomeric cyclo(leu-Pro) against conidia germination and appressorium formation of *Colletotrichum orbiculare*

We analyzed the three isomeric form of cyclo(Leu-Pro) that affected germination and appressorium formation. As a result of variance analysis, there are significantly difference in germination rate and appressorium formation between treatments, including control, extract of BC42 and isomers (Supplementary Table 1), additionally, concentrations of DD and LL-form of isomer exhibited significance in germination rate, appressorium formation, and lesion area (Supplementary Table 1). Whereas, there was not statistical difference in germination and appressorium formation by concentrations of DL-form (Supplementary Table 1).

**Figure 3:**
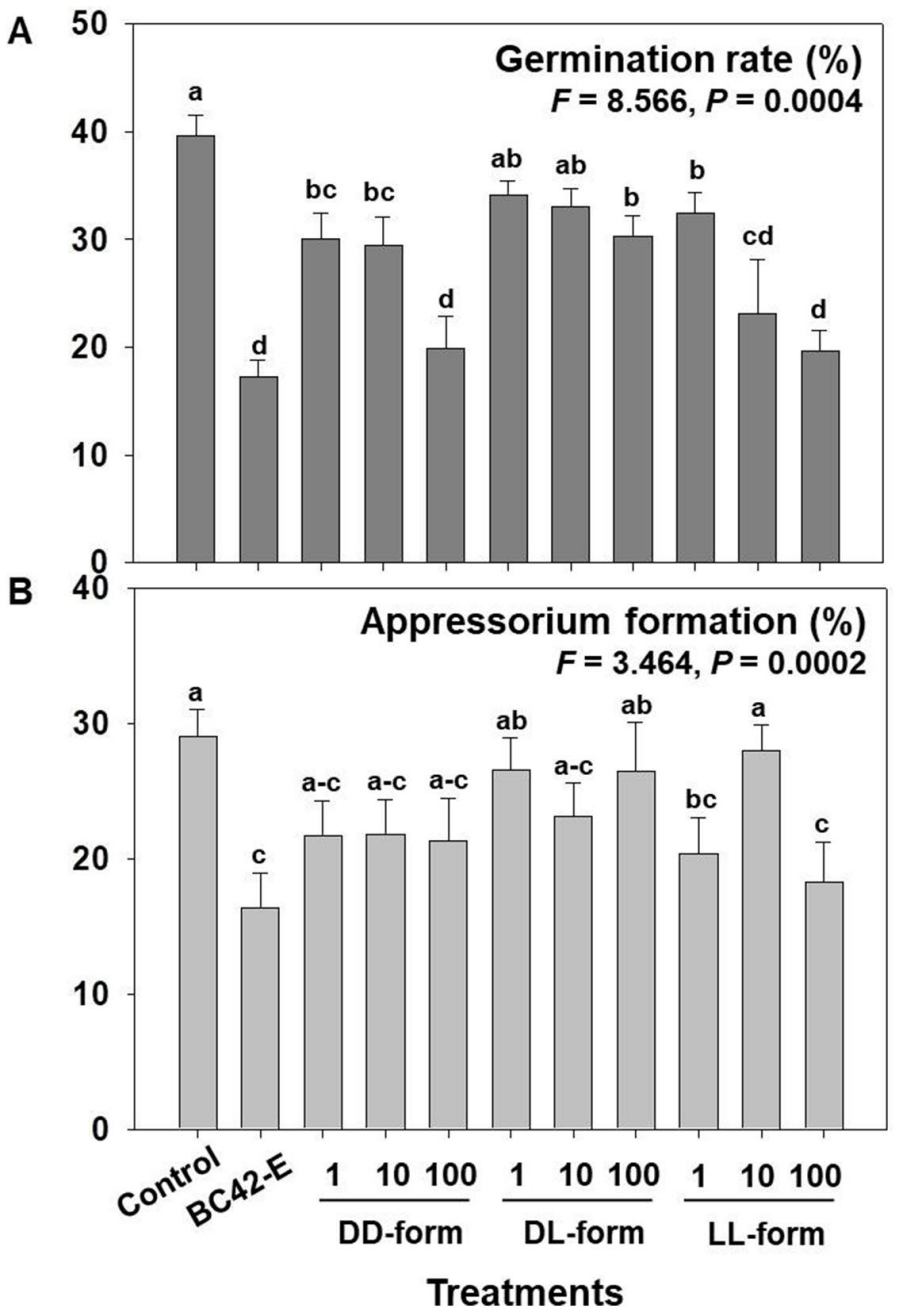
Assessment of the germination rate **(A)** and appressorium formation **(B)** depending on concentration and isomer of cyclo(Leu-Pro). Control: distilled water; BC42-E: 100 μg/mL of ethyl acetate extract from *P. sesami* BC42; DD-form: cyclo(d-Leu-d-Pro); DL-form: cyclo(d-Leu-l-Pro); LL-form: cyclo(l-Leu-l-Pro). Letters on the vertical bar indicate statistical difference assessed by Tukey’s HSD test (*p < 0.05); error bars represent standard error.

The treatments of all tested concentrations of cyclo(d-Leu-d-Pro) (DD-form) and cyclo(l-Leu-l-Pro) (LL-form), and 100 μg/mL of the cyclo(d-Leu-l-Pro) (DL-form) had significantly inhibitory activity against conidial germination of *C. orbiculare* compared to control (Figure 3A). Especially, the 100 μg/mL of DD-form and LL-form exhibited an inhibition rate similar to that of the BC42-extract, at 19.9 and 19.7%, respectively (Figure 3A). In case of DL-form, only 100 μg/mL inhibited germination rate (Figure 3A). Despite germination inhibitory activity of the isomers, only LL-form at concentrations of 1 and 100 μg/mL had an adverse impact on appressorium formation compared to control; the inhibitory activity of LL-form at these concentrations was similar to that of BC42-extract (Figure 3B), while DD and DL-forms did not affect appressorium formation (Figure 3B).

### 3.3. Detached leaf assay of *Colletotrichum orbiculare*

**Figure 4:**
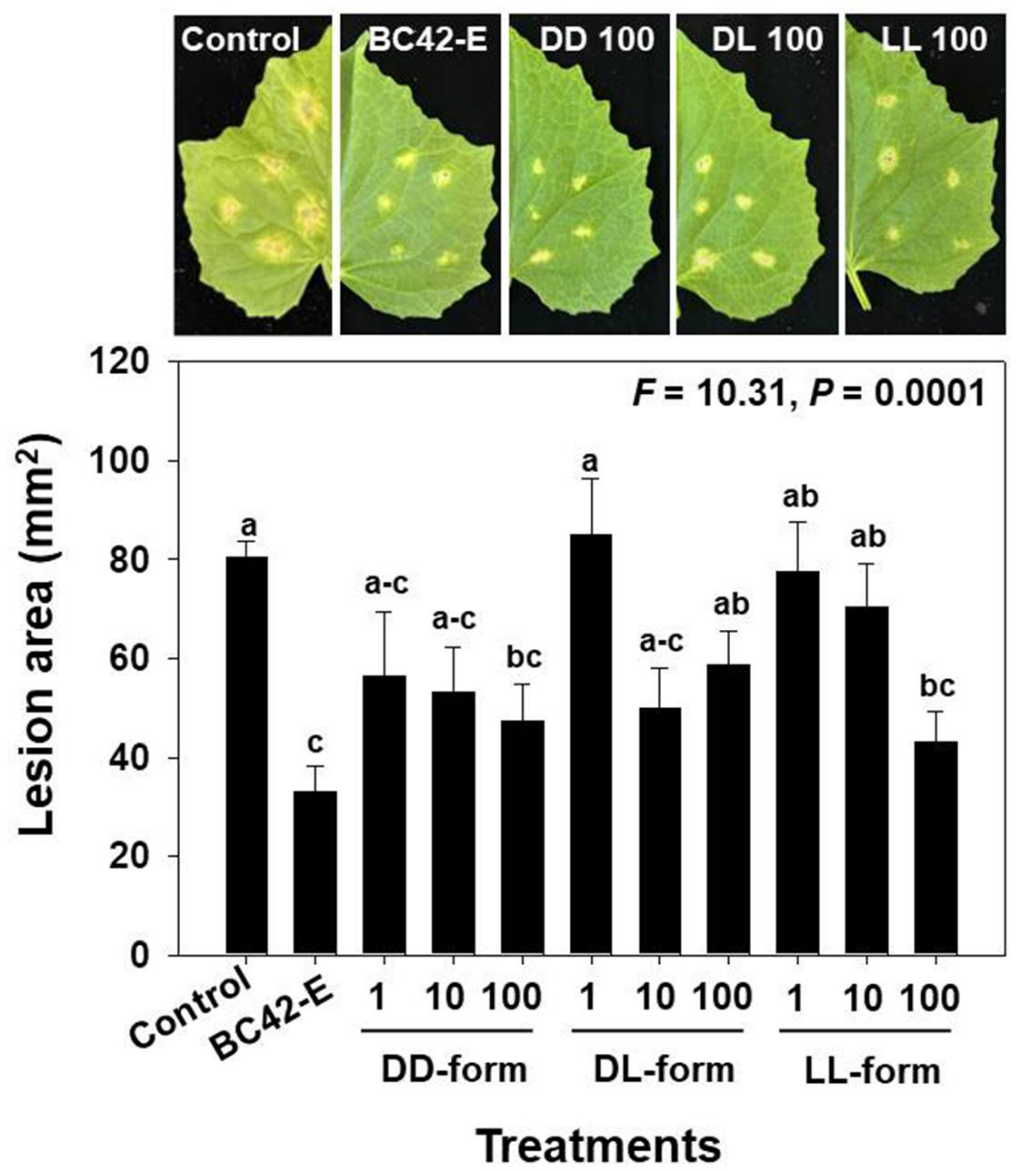
The lesion area following inoculation of *C. orbiculare*, depending on different concentrations of cyclo(Leu-Pro). Control: distilled water; BC42-E: 100 μg/mL of ethyl acetate extract from *Pseudomonas* sp. strain BC42; DD-form: cyclo(d-Leu-d-Pro); DL-form: cyclo(d-Leu-l-Pro); LL-form: cyclo(l-Leu-l-Pro). Letters on the vertical bar indicate statistical difference assessed by Tukey’s HSD test (*p < 0.05); error bars represent standard error.

Detached cucumber leaves treated with *C. orbiculare* conidia in water, BC42 extract, or three isomers of cyclo(Leu-Pro) exhibited typical lesions. As a result of variance analysis, the lesion area in the detached leaf assay was significantly (*p* < 0.001) affected by the concentrations of each isomer (Supplementary Table 1). The lesion area (mm^2^) of *C. orbiculare* on leaves mixed with sterile distilled water was approximately 76.6 mm^2^ at 7 dpi, whereas the BC42-extract exhibited reduction in lesions with an area of 33 mm^2^. All tested concentrations of DD-from had significantly smaller lesion sizes than those of the controls; the lesion areas in detached leaf of cucumber plants treated with 1, 10, and 100 μg/mL DD-form were 56.5 mm^2^, 53.2 mm^2^ and 47.4 mm^2^, respectively (Figure 4). Significantly smaller lesion areas in relation to the control were observed for the DL-form at working concentrations of 10 and 100 μg/mL by 49.9 mm^2^ and 58.7 mm^2^, respectively, while only the LL-form at concentration of 100 μg/mL led to a notable reduction in lesion area by 43.1 mm^2^ compared to the control (Figure 4).

### 3.4. Effects of the isomers on antifungal activity and disease suppression differentially expressed results

**Figure 5:**
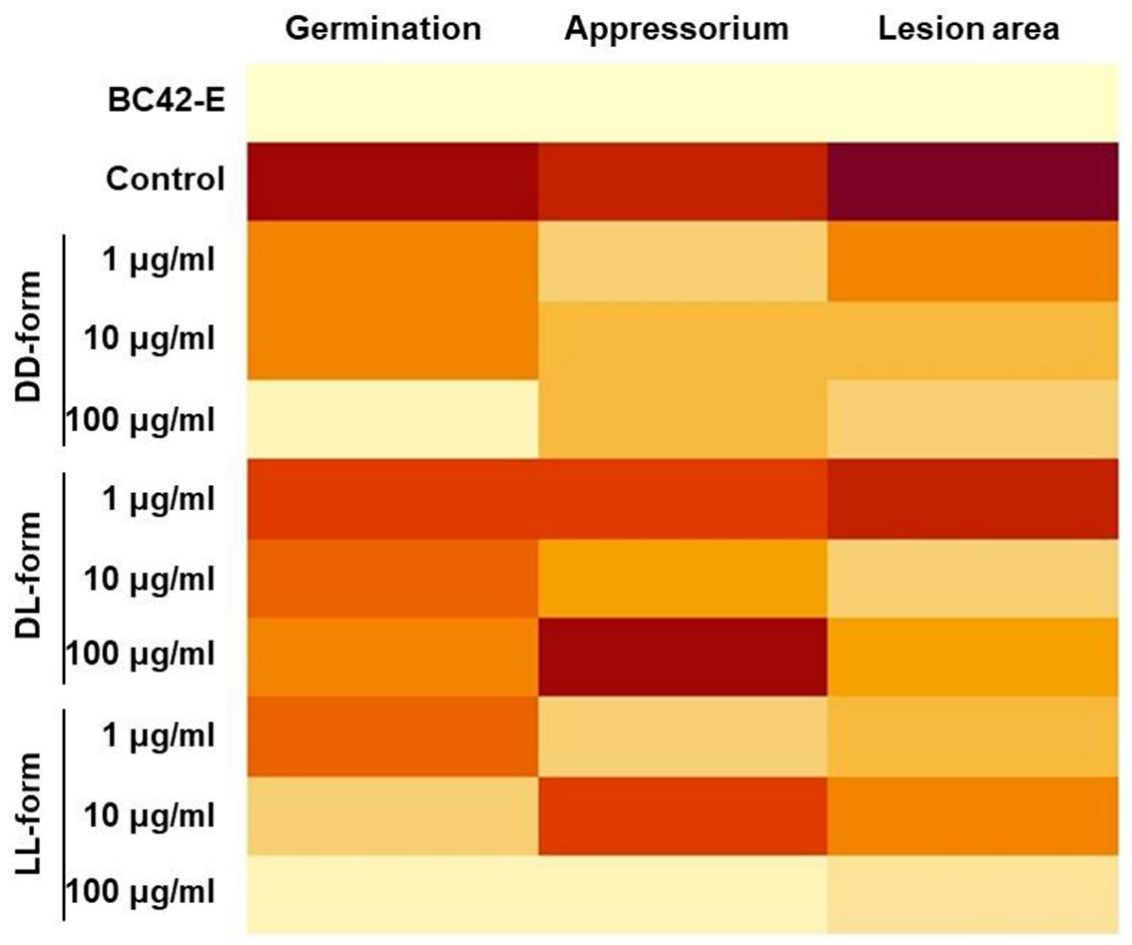
Heatmap of the differentially expressed results in germination, appressorium formation, and lesion area compared with each treatment. Dark red (the higher value) ~ light yellow (the lower value). A heatmap was constructed using the average of each treatment.

**Figure 6:**
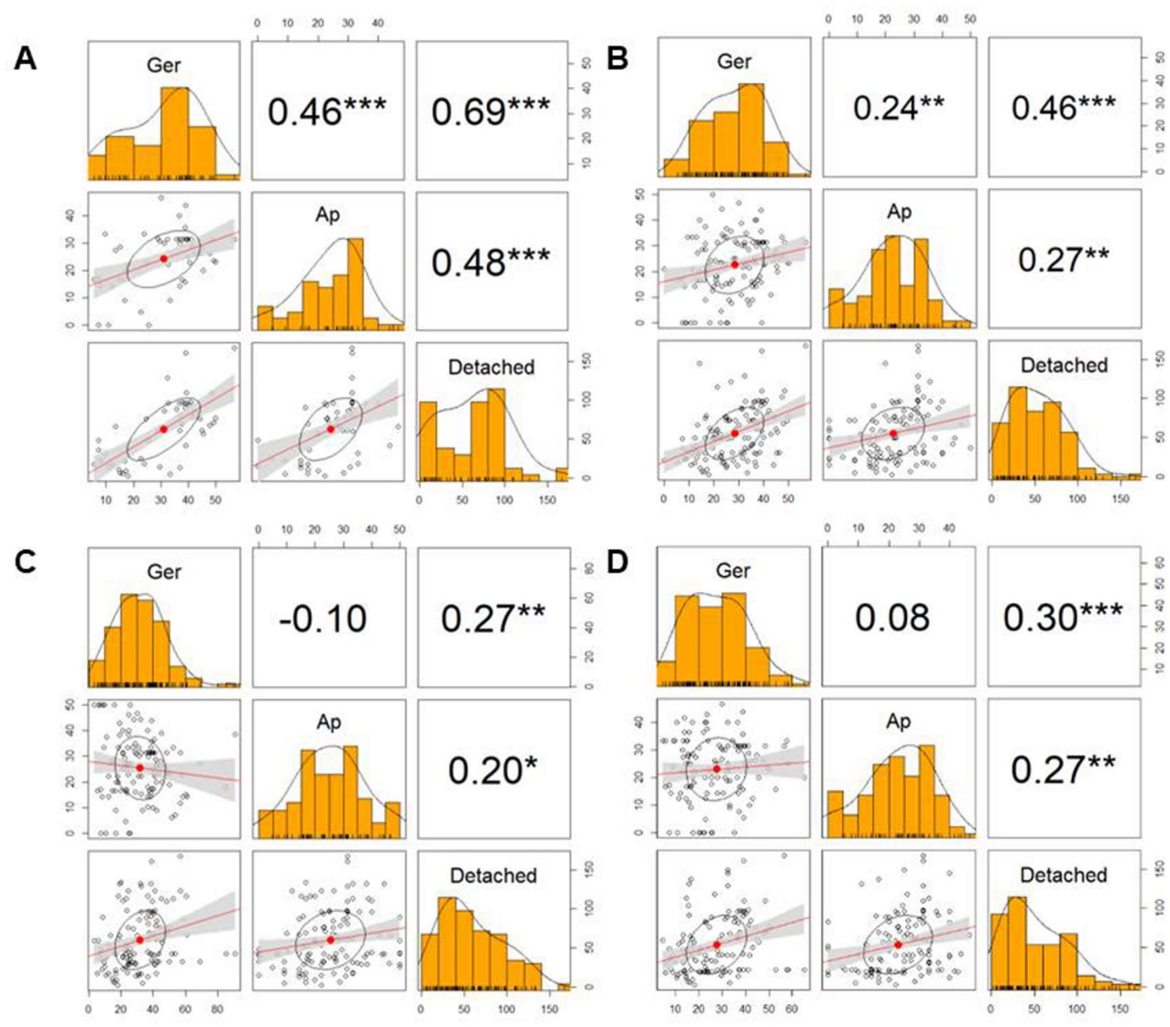
Correlation matrix showing the size of lesion area (Detached) and ability of germination (Ger) and appressorium formation (Ap). In the correlation matrix, the values are correlation coefficients (*r*), *** *p* < 0.001, ** *p* < 0.01, * *p* < 0.05. **(A)**, BC42-E: 100 μg/mL of ethyl acetate extract from *Pseudomonas* sp. strain BC42; **(B)**, DD-form: cyclo(d-Leu-d-Pro); **(C)**, DL-form: cyclo(d-Leu-l-Pro); **(D)**, LL-form: cyclo(l-Leu-l-Pro).

The heatmap of Figure 5 showed differential activity with various colors from a dark red (higher value) to bright yellow (lower value). The BC42-extract showed a bright yellow color in germination, appressorium and lesion area, and only LL-form at concentration of 100 μg/mL showed similar pattern to BC42-extract (Figure 5). In case of DD-form, only concentration of 100 μg/mL had a bright yellow color in lesion area (Figure 5). As a correlation analysis, the lesion area and two factors, including germination and appressorium formation, to be positively interacted (Figure 6). A strong correlation between the lesion area and germination/appressorium formation of *C. orbiculare* in the treatment of BC42-extract was observed, as *r*-values ranged from 0.51 to 0.81 (*p* < 0.001) for germination and from 0.24 to 0.67 (*p* < 0.001) for appressorium formation (Figure 6A). The relationship between lesion area and other CDP forms was examined using Pearson’s correlation, which showed correlations between lesion area and different concentrations of three types of CDPs: DD-form, germination, appressorium (*r* = 0.46 and 0.27 at *p* < 0.001, respectively) (Figure 6B); DL-form, germination, appressorium (*r* = 0.27 at *p* < 0.001 and 0.20 at *p* < 0.05) (Figure 6C); LL-form, germination, appressorium (*r* = 0.30 at *p* < 0.001 and 0.27 at *p* < 0.05, respectively) (Figure 6D).

## 4. Discussion

Antifungal metabolites derived from various bacterial strains have been extensively investigated owing to the pressing need for more ecofriendly and sustainable agriculture strategies. A previous study demonstrated that a filtrate of *P. sesami* BC42 exhibits antifungal activity against *C. orbiculare*. When PDA was amended with the ethyl acetate extract of BC42, mycelial growth and sporulation decreased by 65 and 85%, respectively (Kim et al., 2022).

Antimicrobial peptides have gained increased attention as potential alternatives to overcome the issue of bacterial resistance to conventional antibiotics, and cyclic peptides with antifungal activities have been comprehensively studied. Mika et al. (2011) reported that cyclic peptides exhibit antimicrobial activity, as they can penetrate the lipid bilayer of the cell membrane, disrupt the structure of the membrane, and modify its permeability. In this study, we characterized the BC42-producing substance to be a cyclic peptide, cyclo(Leu-Pro). Cyclo(Leu-Pro) is a linear dipeptide folded from head to tail, and it has a wide range of biological activities, such as antibacterial, antifungal, and antiviral properties (Jamal et al., 2017). The cyclo(Leu-Pro) of *Streptomyces* sp. KH-614 has been found to be effective against vancomycin-resistant enterococci and pathogenic fungi, such as *Pyricularia oryzae* and *Trichophyton rubrum* (Rhee, 2002). Mortality and egg hatching of *Meloidogyne incognita* were inhibited by cyclo(l-Pro-l-Leu) from *P. putida* MCCC 1A00316 (Zhai et al., 2019). *Ganoderma boninense* and *Candida albicans* growth was similarly attenuated by cyclo(Leu-Pro) isolated from the culture medium of *Lactobacillus plantarum* (Kim et al., 2019). In addition, cyclo(l-Pro-l-Leu) produced by *P. putida* WCS358 can activate biosensors for quorum sensing mechanisms (Degrassi et al., 2002).

The initial germination and appressorium formation of conidia are crucial processes during the infection stage (Osherov and May, 2001). We have previously reported that BC42 extracts affect sporulation, appressorium formation, and the mitogen-activated protein kinase signaling pathway (Kim et al., 2022). The cyclo(l-Pro-l-Phe) produced by *E. coli* GZ-34 effectively inhibited the spore formation in *Magnaporthe grisea*, which is a vital pathogenic process of this fungal pathogen (Song et al., 2018). Schwinges et al. (2019) demonstrated that the bifunctional peptide DS01-THA inhibited the formation of *Phakopsora pachyrhizi* appressoria *in vitro*. CDPs produced by *Pseudomonas fluorescens* are effective against grain mold fungi (Begum et al., 2014). When a conidial suspension of *C. orbiculare* was mixed with the BC42 extract, appressorium formation was reduced, and the extract was shown to reduce lesions when sprayed on cucumber leaves (Kim et al., 2022). In our present work, we compared the activities of the three isomers: cyclo(d-Leu-l-Pro), cyclo(d-Leu-d-Pro), and cyclo(d-Leu-l-Pro). We found that they are affected by different forms and concentrations (Supplementary Table 1). Several studies related to bioactivities depending on stereoisomers of CDPs have been performed. Chirality is critical in biological systems because enantiomers frequently exhibit highly different physiological characteristics (Brandl and Deber, 1986). The LL-form at concentration of 100 μg/mL and the BC42-extract displayed similar inhibitory activities in germination, appressorium formation, and detached leaf assays, while the DL-form appeared to be only marginally higher than the DD-form. This may be due to the motion of cyclo(l-Leu-l-Pro) being less restricted within the proline ring, in comparison to that of cyclo(d-Leu-l-Pro), which may at least partially account for the observed variations in activity (Deslauriers et al., 1976). The cyclo(l-Pro-l-Leu) form produced by *Achromobacter xylosoxidans* has been reported to remarkably inhibit the production of highly toxic, carcinogenic, and teratogenic aflatoxins by *Aspergillus parasiticus*, more so than other isomers (Yan et al., 2004). Both diastereoisomer cyclo(l-Pro-l-Tyr) and cyclo(d-Pro-l-Tyr) showed antibacterial activity against *Xanthomonas axonopodis* pv. *citri* and *Ralstonia solanacearum*, with a MIC of 31.25 μg/mL (Wattana-Amorn et al., 2016). Rosetti et al. (2022) reported that the stereoisomer types of cyclo(Leu-Phe) showed antimicrobial activity against *Staphylococcus aureus* with a slight difference in MIC: 25 μg/mL of cyclo(l-Lue-l-Phe), cyclo(l-Lue-d-Phe), and cyclo(d-Phe-l-Phe), and 12.5 μg/mL of cyclo(d-Lue-l-Phe). As mentioned above, CDPs showed differential bioactive effects depending on the isomer structure, and we also found that the LL-form had antifungal and biocontrol activities, and DD-form had a part of germination inhibitory activity. To the best of our knowledge, this is the first time that the LL-form among the stereoisomers of cyclo(Leu-Pro) produced by *P. sesami* BC42 can inhibit the development of *C. orbiculare* as well as disease suppression in cucumber.

Overall, this study has highlighted the differential biocontrol activity of isomeric cyclo(Leu-Pro) compounds isolated from *P. sesami* BC42 against *C. orbiculare*. Our findings indicate that the LL-form of three isomeric cyclo(Leu-Pro) compounds produced by the strain BC42 plays a crucial role in reducing germination and appressorium formation in *C. orbiculare*, these effects showed strong correlations with the lesion area. The results have shown the potential of cyclo(l-Lue-l-Phe) as an antifungal agent, suggesting its suitability for use in sustainable agricultural strategies aimed at protecting crops from infections caused by fungal pathogens.

## Data availability statement

The original contributions presented in the study are included in the article/Supplementary material, further inquiries can be directed to the corresponding author.

## Author contributions

JK and MS contributed to conceptualization, resources, writing—review and editing. JK and J-CK contributed to methodology and validation. JK contributed to data curation and writing—original draft preparation. MS contributed to supervision, project administration, and funding acquisition. All authors have read and agreed to the published version of the manuscript.

## Funding

This work was supported by the National Institute of Agricultural Sciences (No. PJ01497802), Rural Development Administration, Republic of Korea, as well as the 2020-2023 collaborative research program between university and Rural Development Administration, Republic of Korea.

## Conflict of interest

The authors declare that the research was conducted in the absence of any commercial or financial relationships that could be construed as a potential conflict of interest.

## Publisher’s note

All claims expressed in this article are solely those of the authors and do not necessarily represent those of their affiliated organizations, or those of the publisher, the editors and the reviewers. Any product that may be evaluated in this article, or claim that may be made by its manufacturer, is not guaranteed or endorsed by the publisher.

## References

[ref1] Agrios G. (2005). Plant pathology. 5th Amsterdam: Elsevier Academic Press

[ref2] Backer R. Rokem J. S. Ilangumaran G. Lamont J. Praslickova D. Ricci E.. (2018). Plant growth-promoting rhizobacteria: context, mechanisms of action, and roadmap to commercialization of biostimulants for sustainable agriculture. Front. Plant Sci. 9:1473. doi: 10.3389/fpls.2018.01473, PMID: 30405652 PMC6206271

[ref3] Balachandra C. Padhi D. Govindaraju T. (2021). Cyclic dipeptide: a privileged molecular scaffold to derive structural diversity and functional utility. ChemMedChem 16, 2558–2587. doi: 10.1002/cmdc.202100149, PMID: 33938157

[ref4] Begum A. S. Basha S. A. Raghavendra G. Kumar M. V. N. Singh Y. Patil J. V.. (2014). Isolation and characterization of antimicrobial cyclic dipeptides from *Pseudomonas fluorescens* and their efficacy on sorghum grain mold fungi. Chem. Biodivers. 11, 92–100. doi: 10.1002/cbdv.201300045, PMID: 24443429

[ref5] Brandl C. J. Deber C. M. (1986). Hypothesis about the function of membrane-buried proline residues in transport proteins. Proc. Natl. Acad. Sci. U. S. A. 83, 917–921. doi: 10.1073/pnas.83.4.917, PMID: 3456574 PMC322981

[ref6] Damm U. Cannon P. F. Liu F. Barreto R. W. Guatimosim E. Crous P. W. (2013). The *Colletotrichum orbiculare* species complex: important pathogens of field crops and weeds. Fungal Divers. 61, 29–59. doi: 10.1007/s13225-013-0255-4, PMID: 30893003

[ref7] de Mendiburu F. (2019). Agricolae: statistical procedures for agricultural research. Available at: https://CRAN.R-project.org/package=agricolae (accessed on 1 November 2020)

[ref8] Degrassi G. Aguilar C. Bosco M. Zahariev S. Pongor S. Venturi V. (2002). Plant growth-promoting *Pseudomonas putida* WCS358 produces and secretes four cyclic dipeptides: cross-talk with quorum sensing bacterial sensors. Curr. Microbial. 45, 250–254. doi: 10.1007/s00284-002-3704-y, PMID: 12192521

[ref9] Deslauriers R. Grzonka Z. Walter R. (1976). Influence of D and L amino-acid residues on the conformation of peptides in solution: a carbon-13 nuclear magnetic resonance study of *cyclo*(prolyl-leucyl). Biopolymers 15, 1677–1683. doi: 10.1002/bip.1976.360150905, PMID: 963256

[ref10] Dewanjee S. Gangopadhyay M. Bhattacharya N. Khanra R. Dua T. K. (2015). Bioautography and its scope in the field of natural product chemistry. J. Pharm. Anal. 5, 75–84. doi: 10.1016/j.jpha.2014.06.002, PMID: 29403918 PMC5761477

[ref11] Dimkić I. Janakiev T. Petrović M. Degrassi G. Fira D. (2022). Plant-associated *Bacillus* and *Pseudomonas* antimicrobial activities in plant disease suppression via biological control mechanisms - a review. Physiol. Mol. Plant Pathol. 117:101754. doi: 10.1016/j.pmpp.2021.101754

[ref12] Ghajar F. Holford P. Cother E. Beattie A. (2006). Effects of ultraviolet radiation, simulated or as natural sunlight, on conidium germination and appressorium formation by fungi with potential as mycoherbistats. Biocontrol Sci. Tech. 16, 451–469. doi: 10.1080/09583150500532642

[ref13] Houston D. R. Shiomi K. Arai N. Ōmura S. Peter M. G. Turberg A.. (2002). High-resolution structures of a chitinase complexed with natural product cyclopentapeptide inhibitors: mimicry of carbohydrate substrate. Proc. Natl. Acad. Sci. U. S. A. 99, 9127–9132. doi: 10.1073/pnas.132060599, PMID: 12093900 PMC123105

[ref14] Jamal Q. Cho J. Y. Moon J. H. Munir S. Anees M. Kim K. Y. (2017). Identification for the first time of Cyclo(d-pro-l-leu) produced by *Bacillus amyloliquefaciens* Y1 as a nematocide for control of *Meloidogyne incognita*. Molecules 22:1839. doi: 10.3390/molecules22111839, PMID: 29077011 PMC6150376

[ref15] Keswani C. Singh H. B. García-Estrada C. Caradus J. He Y. W. Mezaache-Aichour S.. (2019). Antimicrobial secondary metabolites from agriculturally important bacteria as next-generation pesticides. Appl. Microbiol. Biotechnol. 104, 1013–1034. doi: 10.1007/s00253-019-10300-8, PMID: 31858191

[ref16] Kim J. Ju H. J. Sang M. K. (2022). Bioactive extract of *Pseudomonas* sp. BC42 suppresses the infection stages of *Colletotrichum orbiculare*. J. Plant Pathol. 104, 1443–1455. doi: 10.1007/s42161-022-01225-9

[ref17] Kim Y. J. Kim J. H. Rho J. Y. (2019). Antifungal activities of *Streptomyces blastmyceticus* strain 12-6 against plant pathogenic fungi. Mycobiology 47, 329–334. doi: 10.1080/12298093.2019.1635425, PMID: 31565468 PMC6758635

[ref18] Kubo Y. Takano Y. (2013). Dynamics of infection-related morphogenesis and pathogenesis in *Colletotrichum orbiculare*. J. Gen. Plant Pathol. 79, 233–242. doi: 10.1007/s10327-013-0451-9, PMID: 25275394

[ref19] Lee D. W. Kim B. S. (2015). Antimicrobial cyclic peptides for plant disease control. Plant Pathol. J. 31, 1–11. doi: 10.5423/PPJ.RW.08.2014.0074, PMID: 25774105 PMC4356600

[ref20] Liu T. B. Lu J. P. Liu X. H. Min H. Lin F. C. (2008). A simple and effective method for total RNA isolation of appressoria in *Magnaporthe oryzae*. J. Zhejiang Univ. Sci. B 9, 811–817. doi: 10.1631/jzus.B0860011, PMID: 18837109 PMC2565745

[ref21] Mercado-Blanco J. Bakker P. A. H. M. (2007). Interactions between plants and beneficial *Pseudomonas* spp.: exploiting bacterial traits for crop protection. Antonie Van Leeuwenhoek 92, 367–389. doi: 10.1007/s10482-007-9167-1, PMID: 17588129

[ref22] Mika J. T. Moiset G. Cirac A. D. Feliu L. Bardají E. Planas M.. (2011). Structural basis for the enhanced activity of cyclic antimicrobial peptides: the case of BPC194. Biochim. Biophys. Acta Biomembr. 1808, 2197–2205. doi: 10.1016/j.bbamem.2011.05.001, PMID: 21586269

[ref23] Osherov N. May G. S. (2001). The molecular mechanisms of conidial germination. FEMS Microbiol. Lett. 199, 153–160. doi: 10.1111/j.1574-6968.2001.tb10667.x, PMID: 11377860

[ref24] Park G. K. Lim J. H. Kim S. D. Shim S. H. (2012). Elucidation of antifungal metabolites produced by *Pseudomonas aurantiaca* IB5-10 with broad-spectrum antifungal activity. J. Microbiol. Biotechnol. 22, 326–330. doi: 10.4014/jmb.1106.06042, PMID: 22450787

[ref25] Patil S. Nikam M. Anokhina T. Kochetkov V. Chaudhari A. (2017). Multi-stress tolerant plant growth promoting *Pseudomonas* spp. MCC 3145 producing cytostatic and fungicidal pigment. Biocatal. Agric. Biotechnol. 10, 53–63. doi: 10.1016/j.bcab.2017.02.006

[ref26] Raio A. Puopolo G. (2021). *Pseudomonas chlororaphis* metabolites as biocontrol promoters of plant health and improved crop yield. World J. Microbiol. Biotechnol. 37:99. doi: 10.1007/s11274-021-03063-w, PMID: 33978868

[ref27] Rhee K. H. (2002). Isolation and characterization of *Streptomyces* sp. KH-614 producing anti-VRE (vancomycin-resistant enterococci) antibiotics. J. Gen. Appl. Microbiol. 48, 321–327. doi: 10.2323/jgam.48.321, PMID: 12682870

[ref28] Rosetti B. Scarel E. Colomina-Alfaro L. Adorinni S. Pierri G. Bellotto O.. (2022). Self-assembly of homo- and hetero-chiral cyclodipeptides into supramolecular polymers towards antimicrobial gels. Polymers 14:4554. doi: 10.3390/polym14214554, PMID: 36365547 PMC9654196

[ref29] Schwinges P. Pariyar S. Jakob F. Rahimi M. Apitius L. Hunsche M.. (2019). A bifunctional dermaseptin–thanatin dipeptide functionalizes the crop surface for sustainable pest management. Green Chem. 21, 2316–2325. doi: 10.1039/C9GC00457B

[ref30] Shtark O. Shaposhnikov A. Kravchenko L. (2003). The production of antifungal metabolites by *Pseudomonas chlororaphis* grown on different nutrient sources. Mikrobiologiia 72, 645–650. PMID: 14679903

[ref31] Song S. Fu S. Sun X. Li P. Wu J. Dong T.. (2018). Identification of cyclic dipeptides from *Escherichia coli* as new antimicrobial agents against *Ralstonia Solanacearum*. Molecules 23:214. doi: 10.3390/molecules23010214, PMID: 29351264 PMC6017746

[ref32] Song Z. Lin Y. Zhang X. Feng C. Lu Y. Gao Y.. (2017). Cyclic RGD peptide-modified liposomal drug delivery system for targeted oral apatinib administration: enhanced cellular uptake and improved therapeutic effects. Int. J. Nanomedicine 12, 1941–1958. doi: 10.2147/IJN.S125573, PMID: 28331317 PMC5354530

[ref33] Team, R (2015). RStudio: Integrated development environment for R. Boston, MA: RStudio. Inc., 14.

[ref34] Team, RDC (2009). A language and environment for statistical computing http://www.R-project.Org.

[ref35] Wattana-Amorn P. Charoenwongsa W. Williams C. Crump M. P. Apichaisataienchote B. (2016). Antibacterial activity of cyclo (L-pro-L-Tyr) and cyclo (D-pro-L-Tyr) from *Streptomyces* sp. strain 22-4 against phytopathogenic bacteria. Nat. Prod. Res. 30, 1980–1983. doi: 10.1080/14786419.2015.1095747, PMID: 26469746

[ref36] Wedge D. Nagle D. G. (2000). A new 2D-TLC bioautography method for the discovery of novel antifungal agents to control plant pathogens. J. Nat. Prod. 63, 1050–1054. doi: 10.1021/np990628r, PMID: 10978195

[ref37] Weller D. M. (2007). *Pseudomonas* biocontrol agents of soilborne pathogens: looking back over 30 years. Phytopathology 97, 250–256. doi: 10.1094/PHYTO-97-2-0250, PMID: 18944383

[ref38] Yan P. S. Song Y. Sakuno E. Nakajima H. Nakagawa H. Yabe K. (2004). Cyclo(L-leucyl-L-prolyl) produced by *Achromobacter xylosoxidans* inhibits aflatoxin production by *aspergillus parasiticus*. Appl. Environ. Microbiol. 70, 7466–7473. doi: 10.1128/AEM.70.12.7466-7473.2004, PMID: 15574949 PMC535151

[ref39] Zhai Y. Shao Z. Cai M. Zheng L. Li G. Yu Z.. (2019). Cyclo(l-pro^−^l-leu) of *Pseudomonas putida* MCCC 1A00316 isolated from Antarctic soil: identification and characterization of activity against *Meloidogyne incognita*. Molecules 24:768. doi: 10.3390/molecules24040768, PMID: 30791605 PMC6412658

