# Supplementary material for: Identification of isomeric cyclo(leu-pro) produced by Pseudomonas sesami BC42 and its differential antifungal activities against Colletotrichum orbiculare
Source: Front Microbiol. 2023 Aug 10;14:1230345. doi: 10.3389/fmicb.2023.1230345 (PMC10448827; doi:10.3389/fmicb.2023.1230345)
Supplement: Supplementary file 1 [file Table_1.docx]

| Factors | Germination | |  | Appressorium | |  | Lesion area | |
| --- | --- | --- | --- | --- | --- | --- | --- | --- |
|  | F-value | P-value |  | F-value | P-value |  | F-value | P-value |
| Treatment | 7.805 | 0.000512 ^***^ |  | 3.708 | 0.02587 ^*^ |  | 2.777 | 0.06408 |
| DD-form | 25.08 | 3.52e-12 ^***^ |  | 4.014 | 0.00963 ^**^ |  | 17.48 | 3.36e-09 ^***^ |
| DL-form | 1.89 | 0.136 |  | 1.537 | 0.21 |  | 11.73 | 1.22e-06 ^***^ |
| LL-form | 23.09 | 1.95e-11 ^***^ |  | 7.147 | 0.000213 ^***^ |  | 16.22 | 1.16e-08 ^***^ |

Supplementary table 1. The analysis of variance results for treatments (Control, BC42E, isomers), and concentrations (0, 1, 10, and 100 μg/ml) of isomers

Asterisks denote significant differences.
